# Cardiopulmonary Exercise Testing: Deciphering Cardiovascular Complications in Systemic Sclerosis

**DOI:** 10.31083/RCM25914

**Published:** 2025-01-21

**Authors:** Ailia Giubertoni, Mattia Bellan, Luca Cumitini, Giuseppe Patti

**Affiliations:** ^1^Division of Cardiology, Maggiore della Carità Hospital, 28100 Novara, Italy; ^2^Department of Translational Medicine, University of Eastern Piedmont, 28100 Novara, Italy; ^3^Division of Internal Medicine, Maggiore della Carità Hospital, 28100 Novara, Italy

**Keywords:** cardiopulmonary exercise testing, pulmonary arterial hypertension, heart failure, systemic sclerosis

## Abstract

Cardiac manifestations in systemic sclerosis (SSc) are variable and are associated with a poor prognosis, frequently resulting in impaired right ventricular function and heart failure. A high proportion of patients with SSc experience pulmonary arterial hypertension (PAH), interstitial lung disease, or myocardial involvement, all of which can lead to exercise intolerance. In this context, cardiopulmonary exercise testing (CPET) is a useful tool for diagnosing exercise intolerance, elucidating its pathophysiology, and assessing its prognosis. CPET can also identify patients with SSc at higher risk of developing PAH. Despite its utility, current guidelines for CPET do not include the evaluation of patients with SSc, nor do standard SSc management guidelines consider CPET in the clinical work-up. This review summarizes the development, supporting evidence, and application of CPET in assessing cardiac involvement in patients with SSc.

## 1. Cardiopulmonary Exercise Testing for Systemic Sclerosis

### 1.1 Introduction

Cardiopulmonary exercise testing (CPET) is a noninvasive technique for assessing functional capacity and characterizing exercise limitations [1], providing critical insights into exercise physiology [2]. To ensure the accuracy of results, CPET should be performed in centers that adhere to stringent standards for conducting the examination and evaluating outcomes [3, 4]. This testing modality not only assesses functional capacity and detects both symptomatic and asymptomatic exercise intolerance [5, 6], but can also determine the severity of the disease and distinguish between different causes of dyspnea and exercise impairment [5, 7]. The indications for CPET in clinical practice have expanded significantly over the last decades [8]. Previously confined primarily to in the management heart failure [9], where it delivers substantial diagnostic and prognostic information [10, 11], its use is now broader, reflecting its growing importance in clinical settings.

Systemic sclerosis (SSc) is a severe connective tissue disease (CTD) inflammatory disease characterized by vascular dysfunction and excessive fibrosis. Its clinical manifestations vary from limited skin involvement to life-threatening effects on internal organs, particularly the heart, kidneys and lungs [12]. The European Clinical Trials and Research Group reports that cardiac causes account for 26% of SSc disease-related mortality [13]. The true prevalence of cardiac involvement in SSc remains elusive, hindered by the absence of a consensus definition and the challenges associated with detecting subclinical cardiac conditions [14].

### 1.2 Pathophysiology of Cardiovascular Involvement in Systemic Sclerosis

**Fig. 1.**
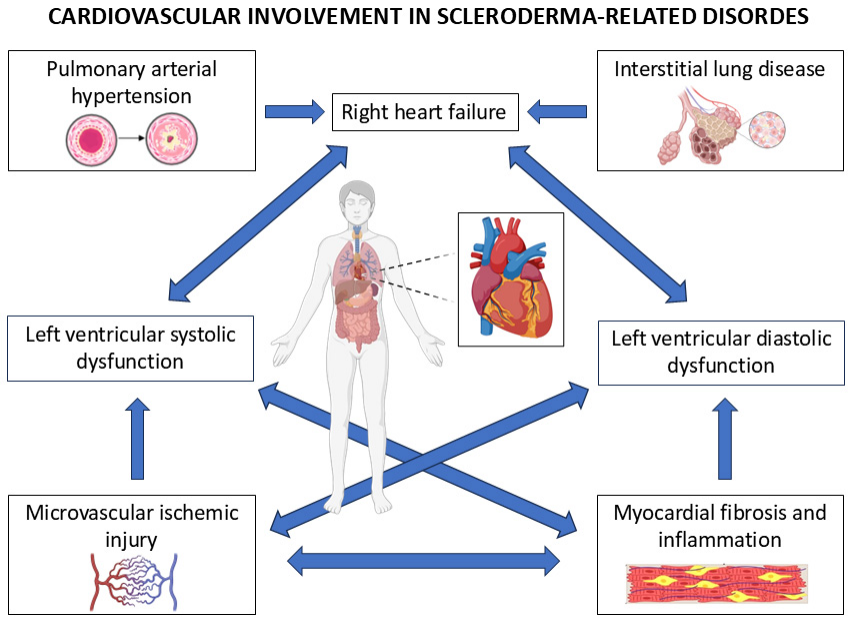
**Pathophysiology of cardiovascular involvement in systemic sclerosis (SSc)**. SSc leads to pulmonary arterial hypertension, interstitial lung disease, microvascular ischemic injury and myocardial fibrosis and inflammation. All these factors interact with each other generating right heart failure and left ventricular systolic and diastolic dysfunction.

There are many cardiovascular manifestations of SSc, which can be categorized as primary or secondary (Fig. 1). Primary manifestations include systolic and diastolic dysfunction, predominantly stemming from myocardial fibrosis, autonomic neuropathy, and small vessel disease affecting the pericardium, myocardium, coronary arteries, valvular structures, and the conduction system [15]. Secondary manifestations are often complications arising from pulmonary arterial hypertension (PAH), interstitial lung disease (ILD), renal failure, amyloidosis, or other systemic complications of SSc [16, 17].

The pathogenesis of primary cardiac involvement is not well understood, and likely encompasses microvascular injury, vasoconstriction, chronic ischemia-reperfusion damage, cardiac inflammation, and fibrosis [17]. These different potential pathophysiological mechanisms may lead to various clinical presentations including myocardial inflammation, myocardial fibrosis, restrictive cardiomyopathy, systolic and/or diastolic ventricular dysfunction, heart failure, valvular regurgitation, coronary artery disease, rhythm and conduction disturbances, and pericardial disease (both effusion and fibrosis) [15].

Primary myocardial involvement in SSc is often clinically occult, yet it carries a poor prognosis when symptomatic [18]. This involvement may result from damage to the microvascular bed, which leads to repeated focal ischemic injuries and irreversible myocardial fibrosis [19], or from a primary systemic myositic disease [20]. The most recognized classical presentation of myocardial involvement in SSc is left ventricular systolic dysfunction [21]. However, diastolic dysfunction, as detected by tissue-doppler echocardiography or speckle-tracking analysis is also prevalent and indicative of a poor prognosis [22, 23]. An observational study of 153 SSc patients identified that 23% exhibited defined left ventricular diastolic dysfunction, a condition predictive of mortality risk [24]. In another study [25], among a cohort of 333 SSc patients, diastolic dysfunction was present in 17% at baseline and increased to 29% over a 3.4-year follow-up, with affected patients experiencing over a 4-fold increase in mortality compared to patients without diastolic dysfunction at baseline.

Notably, PAH is a severe chronic disease characterized by a mean pulmonary artery pressure greater than 20 mmHg and pulmonary vascular resistance exceeding 2 Wood Units. PAH harbors a poor prognosis and frequently results in impaired right ventricular function and heart failure [26]. Patients with SSc are particularly vulnerable to developing PAH, with a prevalence of SSc-associated PAH ranging from 5% to 12% [27, 28, 29]. Moreover, many patients with SSc exhibit ILD, with or without accompanying PAH [30]. This ILD can further complicate the clinical picture by contributing to myocardial involvement and diastolic dysfunction [31].

Collectively, these observations underscore the significant prevalence of clinical or subclinical cardiac involvement in SSc patients, which often results in exercise intolerance. In this context, CPET emerges as a valuable diagnostic tool, helping to elucidate the pathophysiological underpinnings of these cardiac issues. This review aims to summarize the development and the evidence supporting the application of CPET in assessing heart involvement in patients with SSc.

## 2. Literature Review

### 2.1 Exercise Intolerance and Differential Diagnosis of Cardiovascular Causes of Functional Impairment in SSc

Exercise intolerance is a common complication in SSc, primarily driven by myocardial involvement, PAH, ILD or overlap between these conditions. CPET offers an objective and quantitative evaluation of a subject’s functional capacity. This testing enables a deeper understanding of how various cardiopulmonary diseases individual impact exercise performance [11].

In 1996, Schwaiblmair *et al*. [32] conducted CPET on 78 patients with SSc (mean duration of disease of 8.2 years), 43% of whom were symptomatic for exertion dyspnea. The patients exhibited a reduced maximum exercise capacity, including a slight decline in maximum oxygen consumption (VO_2_ max) and anaerobic threshold (AT). Notably, those with diffuse cutaneous SSc experienced significantly lower VO_2_ max levels. Moreover, they reported an increased physiological dead space over tidal volume ratio (Vd/Vt) and an increase in alveolar-arterial oxygen (O_2_) difference at end-exercise [P(A-a)O_2_], potentially indicative of changes in the alveolar epithelium mainly due to alveolitis, fibrosis, and pulmonary edema. The authors concluded that CPET findings differ between patients with diffuse and limited skin involvement, and that in patients with lung disease, both the Vd/Vt and the P(A-a)O_2_ increase during exercise.

Sudduth *et al*. [33] reached similar conclusions analyzing a small cohort of 15 patients with SSc. Among the cohort, 11 patients completed CPET and exhibited low O_2_ pulse (5.1 ± 0.4 mL O_2_/beat) and reduced O_2_ consumption at AT (VO_2_ at AT/VO_2_ max predicted 0.21 ± 0.02), consistent with circulatory impairment during exercise. Among these, nine were predominantly limited by circulatory issues, and four of these had pulmonary vascular disease, as indicated by impaired gas exchange. In 2 of these 11 patients arterial oxygen desaturation was present. These findings suggest that in SSc patients capable of maximal effort, circulatory impairment appears to be more important in reducing exercise tolerance than lung involvement.

More recently Cuomo *et al*. [34] studied 46 SSc patients undergoing exercise, along with 23 sex and age matched healthy volunteers. Patients with SSc exhibit significantly compromised cardiovascular and respiratory metrics during exercise, as evidenced by a a significantly lower VO_2_ max. Specifically, 93.5% of patients have a VO_2_ max below 80% of the predicted value, with a mean of 58.44% ± 12.28 in SSc patients versus 89.78% ± 8.19 in volunteers (*p* < 0.0001). Additionally, these patients demonstrated a significantly decreased O_2_ pulse (7.21 mL/beat ± 1.54 versus 12.24 mL/beat ± 3.06, *p* < 0.0001), a significantly reduced ventilatory volume (43.65 L/min ± 10.77 versus 63.51 L/min ± 20.66, *p* < 0.0001), and a significantly lower AT in relation to the predicted VO_2_max (39.83% ± 8.68 versus 58.31% ± 6.5, *p* < 0.0001). Regression analysis revealed a significant correlation between impaired exercise performance (expressed as VO_2_ max adjusted for body weight) and the severity of heart involvement (*p* = 0.001), left ventricular diastolic dysfunction (*p* = 0.009), severity of lung involvement (*p* = 0.013), and Health Assessment Questionnaire Disability Index score (*p* = 0.016).

Yiu *et al*. [23] demonstrated that subtle myocardial dysfunction, assessed by speckle tracking strain analysis, is associated with lower functional capacity. In a study assessing 113 SSc patients, the authors found that subtle left ventricular contraction abnormalities could be detected before any changes were noted in routine global echocardiographic parameters. These early contraction abnormalities were found to be independently associated with patient’s functional capacity as evaluated by CPET. The study further demonstrated significant correlations between left ventricle global longitudinal and circumferential strains and the predicted VO_2_ max, independent of age, SSc subtype, and lung function. This observation thus provides direct evidence that even early-stage, subclinical left ventricular systolic dysfunction significantly contributes to functional capacity impairment in SSc. These findings suggest that impaired exercise performance in SSc is a multifactorial condition.

To date, evidence for CPET to distinguish among the various causes of exercise impairment in SSc remains limited. In a small study involving 19 SSc patients, Walkey *et al*. [35] attempted to assess if CPET in conjunction with right-heart catheterization (RHC), could differentiate between different sources of exercise limitation. These included pulmonary vascular limitations, left ventricular diastolic dysfunction, ventilatory limitations (such as restrictive lung disease), or deconditioning/cardiovascular limitation. Patients with pulmonary vasculopathy showed reduced maximal VO_2_ and AT, along with a high ventilatory equivalent for carbon dioxide (VE/VCO_2_), without evidence of pulmonary venous hypertension during RHC, demonstrated by a pulmonary capillary wedge pressure (PCWP) below 18 mmHg. Conversely, diastolic dysfunction was identified in patients with analogous CPET findings, echocardiographic documentation of preserved ejection fraction, and RHC indicating exercise peak PCWP at or above 18 mmHg and a pulmonary artery diastolic pressure to-PCWP gradient 5 mmHg or less. Ventilatory limitation were identified in patients with a low VO_2_ peak and a limited breathing reserve. Cardiovascular dysfunction was suspected in cases of a low VO_2_ peak without signs of ventilatory, pulmonary vascular, or left ventricular impairment at CPET or RHC, coupled with abnormalities on a resting echocardiogram. Finally, deconditioning was defined when other causes of exercise limitation were ruled out, but CPET still demonstrated a low peak VO_2_. Each of these different causes of exercise limitation were represented in the study population. The authors conclude that traditional methods such as physical examination, pulmonary function test (PFT), imaging, or echocardiography alone are insufficient to reliably predict the etiology of exercise limitation, which can be more accurately determined through the combined use of CPET/RHC.

Dumitrescu *et al*. [36] attempted to determine if CPET is able to distinguish signs of pulmonary vasculopathy from other causes of exercise limitation in a relatively larger population of 30 SSc patients). However, only patients without previously diagnosed cardiac or pulmonary impairment were included in the study cohort, making the classification process for distinguishing the different causes of exercise limitation less clear.

A larger population of SSc patients evaluated by CPET was described by Boutou *et al*. [37] in 2016. They assessed the prevalence and potential causes of limited exercise capacity in a population of 78 clinically stable SSc patients with perceived exertional dyspnea or reduced physical performance. They categorized patients into 4 groups: normal exercise capacity or subnormal exercise capacity (not limited by evident heart or lung disease), patients with respiratory limitations, those with left ventricular dysfunction, and finally those with pulmonary vasculopathy. Table 1 (Ref. [37]) summarizes the 4-gropus categorization of SSc patients.

**Table 1.** **Categorization of SSc patients based on CPET parameters: normal or subnormal exercise capacity, respiratory limitation, left ventricular dysfunction or pulmonary vasculopathy**.

Normal or subnormal exercise capacity	Respiratory limitation
-normal peak VO_2_ (≥80% predicted) or low peak VO_2_ (<80% predicted)	-low BR (<11 liters)
-normal AT (≥75% predicted)	irrespective of peak VO_2_, AT, VE/VCO_2_ and SPO_2_ values
-normal BR (≥11 liters)	
-normal VE/VCO_2_ at AT (<34)	
-no resting hypoxemia (rest arterial oxygen saturation ≥95%)	
-no exercise-induced hypoxemia (rest SPO_2_ – peak SPO_2_ ≤4%)	
Left ventricular dysfunction	Pulmonary vasculopathy
-reduced peak VO_2_ (<80% predicted)	-reduced peak VO_2_ (<80% predicted)
-reduced AT (<75% predicted)	-reduced AT (<75% predicted)
-normal or high VE/VCO_2_ at AT	-high VE/VCO_2_ at AT (<34)
-increasing or neutral ΔPetCO_2_	-decreasing ΔPetCO_2_
-no resting and no exercise-induced hypoxemia	Or
	-normal peak VO_2_ (≥80% predicted)
	-normal AT (≥75% predicted)
	-abnormal VE/VCO_2_ at AT (≥34)
	-decreasing ΔPetCO_2_

VO_2_, oxygen consumption; AT, anaerobic threshold; BR, breathing reserve; VE, minute ventilation; VCO_2_, volume of exhaled carbon dioxide; SPO_2_, rest arterial oxygen saturation; PetCO_2_, end-tidal partial pressure of carbon dioxide; ΔPetCO_2_, change of PetCO_2_ from rest to AT; CPET, cardiopulmonary exercise testing; SSc, systemic sclerosis. Derived from Boutou *et al*. [37].

In the study, an equal percentage of patients (32.1%) were identified with pulmonary vasculopathy or exhibited normal/subnormal exercise capacity [37]. Additionally, 25.6% presented with left ventricular dysfunction, while 10.2% faced respiratory limitations. Differentiating patients with pulmonary vasculopathy from others proved challenging, as the end-tidal partial pressure of carbon dioxide (PETCO_2_) at peak VO_2_ was not distinctive enough to discriminate it from left ventricular dysfunction. Therefore, differentiation from the latter group relied on observing a decrease in PETCO_2_ from rest to AT. The authors conclude that in patients with SSc, combining CPET gas exchange pattern assessment with baseline measurements can effectively differentiate the causes of exercise limitation.

Recently, the combination of CPET with other diagnostic tools has been defined as “*complex CPET*” [38]. This integrated approach significantly enhances the pathophysiological insights provided by standard CPET. Complex CPET can be categorized into three levels based on the invasiveness of the methods used alongside it: non-invasive complex CPET, minimally invasive complex CPET, and invasive complex CPET. Non-invasive complex CPET may include additions such as non-invasive cardiac output determination, transthoracic echocardiography, thoracic ultrasound or lung diffusion analysis. Minimally invasive complex CPET might incorporate esophageal balloon recordings or serial arterial blood sampling. Invasive complex CPET involves more intrusive procedures, such as the insertion of a Swan-Ganz catheter in the pulmonary artery.

In 2021, Brown *et al*. [39] conducted a study using complex CPET cardiovascular magnetic resonance (CMR)-augmented CPET (CMR-CPET). Their objective was to more accurately determine the causes of exercise intolerance in SSc using CMR-CPET. They compared SSc patients (with and without PAH) to healthy controls and patients with PAH from other causes. This comparison aimed to investigate the specific contributions of SSc and PAH to exercise intolerance. By combining CPET-derived VO_2_ and CMR-derived cardiac output, they calculated the arteriovenous O_2_ content gradient (ΔavO_2_), a marker of tissue oxygen extraction. All patients had a reduced peak VO_2_ compared to healthy subjects (*p* < 0.022). Specifically, SSc patients had low peak ΔavO_2_ compared to healthy controls (*p* < 0.03), even those without PAH. Conversely, all patients with PAH, regardless of their underlying cause, showed reduced peak cardiac output compared to healthy controls and SSc patients without PAH (*p* < 0.006). Additionally, higher hemoglobin levels were associated with higher peak ΔavO_2_ independent of disease type (*p* = 0.025), and higher myocardial T1 was associated with lower peak stroke volume (*p* = 0.011). The study concluded that CMR-CPET provides valuable insights into the underlying causes of exercise intolerance in different types of SSc. In particular, evaluation of peak ΔavO_2_ offers new understanding regarding the role of skeletal muscle in the reduced exercise tolerance among patients with SSc.

### 2.2 Screening for PAH in SSc

PAH associated with CTD, particularly SSc, is reported in 5% to 19% of patients [14, 27, 40]. The prognosis is even worse than that of the idiopathic form of PAH [41]. Furthermore, PAH is a common cause of SSc-related death, accounting for around 30% of deaths among patients with SSc [42]. The survival rate for SSc patients who develop PAH is alarmingly low, with only a 52% three-year survival rate [43]. Early intervention in PAH patients can lead to improved outcomes, whereas delayed treatment is associated with clinical deterioration [44] and a markedly reduced life expectancy compared to those whose PAH is detected early [45]. Thus, early disease detection is crucial for initiating timely and effective therapy, which is the most important prognostic factor [46, 47]. For this reason, the latest guidelines suggest that patients with SSc must be screened annually for the development of PAH [26], underscoring the necessity of vigilant monitoring to manage this severe complication effectively.

The gold standard for the diagnosis of PAH is RHC [26], but there remains a need for reliable non-invasive methods to detect it in early stages. Echocardiography is currently recommended as a screening tool for PAH [26]. However, its effectiveness for early detection in SSc patients with is limited, with a study showing it has only moderate sensitivity (71%) and specificity (69%) [48].

To improve early detection, the Diagnosis and Early Treatment of Pulmonary Arterial HypEnsion ConnECTed to Systemic Sclerosis (DETECT) algorithm was developed, which includes an initial step directing patients to echocardiography based on a composite score from other variables. These variables include forced vital capacity, lung diffusing capacity for carbon monoxide, presence of telangiectasias, presence of anti-centromere antibodies, serum urate levels, N-terminal pro brain-natriuretic-peptide (BNP), and right axis deviation on electrocardiogram. This tool demonstrates a very high sensitivity (96%) in this setting [40], but its specificity is relatively low (48%). Consequently, while the DETECT score efficiently identifies most patients at risk, its low specificity results in a high number of unnecessary RHC [40], indicating a need for more precise non-invasive diagnostic approaches.

The most recent guidelines recommend the use of the DETECT algorithm for SSc patients who have had the disease for at least three years. This strategy is intended to identify asymptomatic patients with PAH and is designated as a class I recommendation [26] Furthermore, the guidelines suggest that assessing the risk of PAH in SSc patients, particularly based on symptoms such as breathlessness, should involve a combination of evaluations. These include echocardiograms, PFTs, and biomarkers like BNP or N-terminal proBNP. This approach is categorized as a class IIa recommendation [26], emphasizing its importance in early detection and management of PAH in this patient population.

In 2017, Dumitrescu *et al*. [49] proposed the use of CPET as a novel tool to better select patients with SSc at higher risk for PAH, potentially necessitating RHC. They enrolled 173 patients with SSc who did not have a confirmed diagnosis of PAH, but were clinically suspected of having the condition, either based on symptoms or pathological findings from non-invasive testing including echocardiography. These patients underwent both CPET on cycle-ergometer and RHC. The study found that RHC confirmed PAH in 48 patients, and CPET parameters significantly correlated with pulmonary hemodynamics. Specifically, peak VO_2_ and VE/VCO_2_ ratio showed the strongest correlations with pulmonary arterial pressure, transpulmonary pressure gradient, and pulmonary vascular resistance. Receiver operating characteristic analysis showed that certain CPET parameters exhibited both high sensitivity and specificity for PAH detection. Notably, peak VO_2_ had a sensitivity of 87.5% and specificity of 74.8%, while the equivalent for carbon dioxide (EQCO_2_) showed a sensitivity of 79.2% and specificity of 82.9%, indicating high diagnostic accuracy. In their cohort, a peak VO_2_ greater than 18.7 mL/kg/min ruled out PAH with a negative predictive value of 1.0. Conversely a nadir VE/VCO_2_ ratio above 45.5 indicated PAH with a positive predictive value of 1.0. Dumitrescu *et al*. conclude that CPET is a valuable non-invasive method for the detection of SSc-associated PAH that may be particularly useful in reducing unnecessary RHC procedures. This approach not only aids in early detection but also enhances the management of patients by potentially avoiding invasive testing where it may not be needed.

Years after the work by Dumitrescu *et al*. [49], both Santaniello *et al*. in 2020 [50] and Bellan *et al*. in 2021 [51] further substantiated the value of CPET in identifying SSc patients at risk for PAH. First, Santaniello’s group enrolled 314 SSc patients, utilizing the DETECT algorithm, (including steps 1 and 2, combined with echocardiography) which identified 96 patients as positive and were referred for CPET prior to RHC. Their findings highlighted the VE/VCO_2_ slope. Their findings highlighted in predicting PAH at RHC, exhibiting a median of specificity 0.778 and positive predictive value of 0.636. Next, Bellan and colleagues [51] recruited 131 patients, including 112 with CTD without PAH, and 8 with CTD and PAH. They also evaluated a cohort of 11 patients with PAH stemming from other etiologies. RHC was performed to confirm the PAH diagnosis in patients suspected of having the condition based on findings from clinical evaluation and echocardiography. In CTD patients, CPET parameters yielding optimal diagnostic results for PAH were: VO_2_ max ≤14.1 mL/kg/min (area under the curve [AUC]: 0.845, 95% confidence interval [CI]: 0.767–0.904, *p* < 0.001), VE/VCO_2_ slope >33.96 (AUC: 0.888, 95% CI: 0.817–0.938, *p* < 0.001) and basal PETCO_2_ ≤27.2 mmHg (AUC: 0.792, 95% CI: 0.709–0.861, *p* < 0.001). A composite score including all three identified parameters demonstrated even better diagnostic performance. Moreover, these parameters were comparable among CTD patients with PAH and patients with PAH of different etiologies. Both studies concluded that CPET could effectively reduce the number of unnecessary invasive procedures and enhance the identification of PAH among SSc patients, demonstrating CPET’s critical role in the non-invasive diagnostic landscape for this patient population.

A recent study from 2023 involving a small cohort of 52 patients with SSc examined PAH screening [52]. The study used cycle-ergometer CPET which suggested PAH in 16 patients. Of these, resting RHC confirmed PAH in 5 patients, while exercise RHC confirmed it in an additional 7 patients. This resulted in a diagnostic sensitivity of 100% when CPET was combined with both rest and exercise RHC. In contrast, the DETECT score identified 10 patients with potential PAH, of whom only 3 were confirmed by RHC, showing a guideline-based diagnostic algorithm sensitivity of 70%. Thus, CPET, in conjunction with exercise RHC, could potentially diagnosis PAH earlier than the established screening tools.

The latest guidelines on pulmonary hypertension (PH) [26] recommend CPET within the diagnostic algorithm to evaluate suspected causes or, once the diagnosis is established, to determine prognosis. The guidelines recommend annual screening for PAH in SSc patients regardless of symptoms, emphasizing that early intervention improves outcomes. The existing screening protocols include combinations of clinical variables, biomarkers, PFT, and echocardiography. Despite the potential of CPET as an insightful and promising tool, its use in screening for PAH in SSc patients is currently recommended only for symptomatic individuals. In such cases, exercise echocardiography, CPET, or CMR may be considered to support the decision-making process regarding the need for RHC. This recommendation is classified as a Class IIb, indicating a less definitive benefit, and level of evidence C, reflecting a lower degree of certainty due to limited data.

A summary of studies evaluating role of CPET in PAH screening is provided in Table 2 (Ref. [49, 50, 51, 52]).

**Table 2.** **Characteristics of CPET in screening for PAH in patients with SSc**.

Study (year)	Study design	Number of patients undergoing CPET	Type of patients	Medications	Significant results
Dumitrescu *et al*. (2017) [49]	Multicenter, prospective	173	SSc	Patients receiving targeted PAH medications for other indications such as digital ulcerations were excluded from the analysis.	Peak VO_2_: sensitivity 87.5%, specificity 74.8% at a threshold level of 13.8 mL/min/kg.
					A peak VO_2_ of 18.7 mL/kg/min excluded PAH with NPV of 1.0.
					VE/VCO_2_ ratio >45.5 has PPV of 1.0 for diagnosis of PAH.
Santaniello *et al*. (2020) [50]	Monocenter, prospective	54	SSc	Not specified.	VE/VCO_2_ slope sensitivity equal to 1.0 in 87% of models, with a median sensitivity of 1.0, specificity of 0.778, PPV of 0.636 and NPV of 1.0 at a threshold level of 39 to detect PAH.
Bellan *et al*. (2021) [51]	Monocenter, prospective	131	120 CTD	6 patients endothelin receptor antagonists;	Peak VO_2_: 87.5% sensitive, 83% specific, 98% NPV and 36% PPV.
			11 PH of other etiologies	5 patients phosphodiesterase 5 inhibitors;	VE/VCO_2_ slope: 87.5% sensitive, 82% specific, 98% NPV and 35% PPV.
				1 patient riociguat;	PetCO_2_: 87.5% sensitive, 71% specific, 98% NPV and 25% PPV.
				1 patient selexipag.	
Sánchez-Aguilera Sánchez-Paulete *et al*. (2023) [52]	Monocenter, prospective	52	Scleroderma-related disorders	Not specified.	CPET plus DETECT: 100% sensitivity, 90% specificity, 75% PPV and 100% NPV.

CPET, cardiopulmonary exercise testing; SSc, systemic sclerosis; CTD, connective tissue disease; PH, pulmonary hypertension; VO_2_, oxygen consumption; VE, minute ventilation; VCO_2_, volume of exhaled carbon dioxide; PPV, positive predictive value; NPV, negative predictive value; PetCO_2_, end-tidal partial pressure of carbon dioxide; PAH, pulmonary arterial hypertension; DETECT, Diagnosis and Early Treatment of Pulmonary Arterial HypEnsion ConnECTed to Systemic Sclerosis.

### 2.3 Prognostic Role of CPET

Several studies have demonstrated the prognostic value of CPET in patients with SSc [53, 54, 55, 56, 57, 58]. In particular, utilizing the peak VO_2_ and VE/VCO_2_ slope enables survival prediction in patients with SSc. In a cohort of 210 patients [53] followed for up to 10 years, the presence of PAH (*p* = 0.007), a 6-minute walking test distance <413 meters (*p* = 0.003), a peak VO_2_ < 15.6 mL⋅kg^-1^⋅min^-1^ and a VE/VCO_2_ slope >35 were all negative prognostic predictors.

Hemelein *et al*. [54] conducted a study to assess CPET’s capacity in monitoring the progression and development of SSc, and its correlation with prognosis. The study prospectively tracked 29 SSc patients with standard follow-up plus CPET for a mean of 3.7 years. The findings indicated that traditional clinical parameters, resting lung function, and echocardiographic measures did not predict the development of endpoints associated with a poor prognosis. However, distinct CPET parameters showed significant prognostic value. Specifically, baseline VO_2_ and VE/VCO_2_ were correlated with a reduction in forced vital capacity, indicating lung function deterioration. The AT was linked to the development of digital ulcers, a common complication in SSc, while VE/VCO_2_ was associated with increases in pulmonary arterial pressure, suggesting that several CPET parameters can discriminate between SSc patients with or without adverse outcomes.

In fact, CPET variables have been shown to predict prognosis in patients with PAH [55]. In particular, VO_2_ has provided useful information for further stratification of patients with PAH [56]. On this basis, the aforementioned CPET parameters (peak VO_2_ and VE/VCO_2_ slope) have been included in the last guidelines on PH, in a three-strata model for comprehensive risk assessment [26]. Despite this, the applicability of CPET’s prognostic value to patients with PAH secondary to SSc remains uncertain. In 2012, Deboeck *et al*. [57] demonstrated that the prognostic utility of CPET variables may differ according to the etiology of PAH. They reported that, in a cohort of 136 patients with PAH, peak VO_2_, VE/VCO_2_ slope, and VE/VCO_2_ at AT were predictive of mortality for the 85 patients with idiopathic PAH but were less accurate for the 51 patients with PAH associated to other diseases, including 19 patients with SSc. These data contrast with findings from a larger population [53] study on 210 SSc patients, 52 of whom had PH, including a subgroup of 38 with PAH. Patients with PH underwent RHC; subgroups analysis confirmed the prognostic strength of CPET parameters across all subgroups. Indeed, the peak VO_2_ and VE/VCO_2_ slope demonstrated prognostic value in all patients with SSc, including those with PH.

The prognostic role of CPET in SSc patients without baseline PH was recently investigated by Bournia *et al*. [58]. This study involved 62 SSc patients who underwent CPET, PFT and echocardiography at baseline, followed by PFTs every three years for approximately a decade. Baseline respiratory peak VO_2_ was able to predict PFT deterioration—a decline in forced vital capacity ≥10% or a combined decline in forced vital capacity of 5%–9% plus a decrease in diffusing capacity of the lungs for carbon dioxide ≥15% during follow-up—after adjusting for age, sex, and smoking status (hazard ratio [HR]: 0.874, 95% CI = 0.779–0.979, *p* = 0.021). Moreover, a lower baseline peak VO_2_ was associated with a higher risk for death (HR = 0.861, 95% CI: 0.739–1.003, *p* = 0.054). Thus, it seems reasonable that even in the absence of baseline PH, CPET parameters may predict clinical deterioration and mortality risk in SSc patients.

## 3. Conclusions

Cardiovascular involvement is prevalent in SSc. Despite this, current guidelines for CPET [2, 8, 59] do not specifically recommend its use for evaluating patients with SSc. Similarly, guideline recommendations for SSc management do not include CPET in the clinical work-up [12, 60, 61]. However, CPET provides a comprehensive evaluation and represents a promising tool for pathophysiological evaluation, screening for complications, risk stratification, and follow-up in SSc patients. Additionally, it can provide valuable endpoints for clinical trials. The potential roles of CPET in the context of SSc are listed in Table 3.

**Table 3.** **Potential roles of CPET in SSc management**.

Aspect of management	Role of CPET
Pathophysiological assessment	Provides insights into cardiovascular and pulmonary function, helping to delineate the extent and nature of disease involvement.
Screening for complications	Identifies early signs of pulmonary hypertension, cardiac involvement, and other causes of reduced exercise capacity which might not be detectable through routine clinical assessments.
Risk stratification	Helps classify patients based on severity and prognosis by evaluating exercise tolerance and physiological responses during exertion.
Monitoring and follow-up	Assesses response to therapy and progression of disease by comparing serial measurements, allowing adjustments in treatment strategy.
Clinical trials	Serves as a valuable endpoint in clinical studies by providing objective, quantifiable data on physiological responses to therapeutic interventions.

CPET, cardiopulmonary exercise testing; SSc, systemic sclerosis.

Therefore, most of the aforementioned results need to be confirmed in larger cohorts of patients and through adequately powered multicenter studies. This is necessary to revise the current indications and to extend the application of CPET to CTD.
